# Circadian Regulation of Synaptic Plasticity

**DOI:** 10.3390/biology5030031

**Published:** 2016-07-13

**Authors:** Marcos G. Frank

**Affiliations:** Department of Biomedical Sciences, Elson S. Floyd College of Medicine, Washington State University Spokane, Pharmaceutical and Biomedical Science Building 213, 412 E. Spokane Falls Blvd., Spokane, WA 99202, USA; marcos.frank@wsu.edu; Tel.: +1-509-368-6747; Fax: +1-509-358-7882

**Keywords:** circadian, clock, plasticity, sleep, state-clock model

## Abstract

Circadian rhythms refer to oscillations in biological processes with a period of approximately 24 h. In addition to the sleep/wake cycle, there are circadian rhythms in metabolism, body temperature, hormone output, organ function and gene expression. There is also evidence of circadian rhythms in synaptic plasticity, in some cases driven by a master central clock and in other cases by peripheral clocks. In this article, I review the evidence for circadian influences on synaptic plasticity. I also discuss ways to disentangle the effects of brain state and rhythms on synaptic plasticity.

## 1. Introduction

Synaptic plasticity can be defined as changes in the strength of existing synapses, changes in synapse number or size, or changes in morphological structures that contain or form synapses (e.g., dendritic spines and synaptic boutons). Traditionally, three physiological factors are recognized to trigger or influence synaptic plasticity in vivo: Waking experience, developmental programs and sleep (neurodegenerative synaptic changes are not included as they reflect pathological processes). Experience-dependent plasticity is triggered by changes in sensory input ranging from olfaction to vision. It has been demonstrated in a wide variety of sensory, motor and higher-order circuits in vertebrates and invertebrates [1,2]. Developmental programs include changes in synapses that operate independently of experience. An example of the latter is the early formation of visual circuits, which occurs according to innate instructions and proceeds even in the absence of vision [3].

The roles of experience and development are increasingly well understood, but the precise role of sleep remains mysterious. This is because the effects of sleep on synaptic plasticity vary across species, brain region and ontogenetic status and are partly determined by the kinds of experience that precede sleep [4]. Consequently, the role of sleep in synaptic plasticity is debated [5,6,7,8]. One possible explanation for these disparate results is that some of the plastic changes ascribed to sleep are instead driven by biological clocks. In the following sections, I summarize the evidence that biological clocks constitute a fourth factor in synaptic plasticity. I also present experiments that may disentangle the effects of brain state and rhythms on synaptic plasticity. This article thus extends and further develops topics I have discussed elsewhere [4]. This includes a more detailed investigation of how circadian processes can alter synapse number or strength [4] (for a discussion of clock mechanisms themselves, see [9]).

## 2. Circadian Rhythms in Synaptic Plasticity: Electrophysiological Measures

In mammals, evoked neuronal responses and the ability to induce Hebbian long-term potentiation (LTP) vary across the circadian day. There are diurnal/nocturnal rhythms in rat and monkey hippocampal excitatory post-synaptic potentials (EPSPs) [10] and hippocampal LTP is easier to induce (or is of greater magnitude) in hippocampal slices obtained from rodents sacrificed in the dark (active) phase (relative to the light phase) [11,12,13,14]. Interestingly, deletion of canonical clock genes (*Per1* and *Bmal*) reduces the magnitude of hippocampal LTP in situ [15,16,17]. These findings indicate that peripheral clocks regulate plasticity in the hippocampus. This is consistent with demonstrations of circadian rhythms in hippocampal kinase activity and hippocampal based learning [18,19]. Although less well studied, there is also evidence of circadian rhythms in excitability elsewhere in the brain. Hanada and Kawamura reported circadian rhythms in rat visual circuits in vivo that were independent of vigilance states and abolished by lesions of the mammalian central clock (suprachiasmatic nucleus: SCN) [20]. Circadian rhythms also appear to regulate neuronal firing rates in several reward circuits in the hypothalamus and noradrenergic neurons in the brainstem [21].

Circadian rhythms in neuronal excitability and activity have also been observed in invertebrates. Electroantennograms recorded in *Drosophila* [22] and the cockroach [23] exhibit a circadian rhythm in the response to specific odorants. This rhythm requires normal clock gene function and is driven from a peripheral clock mechanism resident in olfactory neurons [24]. Similarly, the resting membrane potential of large lateral-ventral neurons (LNv) neurons in *Drosophila* is more depolarized at the end of the night and more hyperpolarized at the end of the day [25,26]. In one study, these rhythms persisted in brain explants obtained from flies kept in constant conditions (continuous darkness: DD) [26].

## 3. Circadian Rhythms in Synaptic Plasticity: Morphological Measures

In insects, circadian rhythms are reported in a number of pre and post-synaptic structures [27,28]. Electron microscopy (EM) studies in Drosophila show that the number and size of synapses in visual centers vary in ways that indicate the presence of a peripheral clock [29]. In Drosophila maintained in a light-dark (LD) cycle photoreceptor synapses on interneurons are more abundant and synaptic terminals are larger during the day than night [30]. Interestingly, terminal size and synapse numbers begin to change several hours before the end of the day and begin to increase again during the night (sleep phase). Similar time of day effects are also reported in axonal and dendrite morphology. The axons of Drosophila interneurons swell at the onsets of the light and dark periods, with a maximum observed at the latter time point [28]. The dendrites of one class of interneuron (L2) are larger at the beginning of the day with a unimodal circadian rhythm [31]. In addition, a component of the Drosophila clock (small LNvs) show a rhythmic change in branching complexity along the day in LD and DD, with more complex branching early in the day in LD (relative to early night) and the same relationship when the flies are kept in DD [32]. Time of day changes in axonal termini are also reported and both dendritic and axonal changes are dependent on rhythmic expression of the GTPase Rho1 [33].

Circadian changes in synaptic structure also occur in the Drosophila flight motor neuron MN5 [34]. The synaptic boutons of this neuron grow in the morning, reach a maximum at midnight and then decrease during the rest of the night. These changes reflect the influence of a biological clock as they persist in DD and are prevented by mutations in clock genes [35]. Moreover, they are unaffected by sleep-deprivation during the early night, synaptic silencing during the morning peak of activity, or complete lack of activity over two LD cycles resulting from decapitation [36]. Further suggestive findings are that synaptic boutons and synapses (based on confocal and EM measurements) are more numerous at midnight compared with midday under LD cycles [34]. In the same synapses, the size and distribution of synaptic vesicles change with a bimodal cycle under LD, with smaller vesicles at the beginning of the day and the night, coincident with moments of more intense locomotion activity [37].

There is also evidence for circadian rhythms in vertebrate synapses. One example is the vertebrate ribbon synapse (RS) [38]. RS are found in many structures including the retina, the pineal gland and the vestibular organ. This type of synapse contains an electron-dense “ribbon” with tethered vesicles [39]. In the pineal gland, the number of ribbons, and sometimes also their size, is larger in the night compared with the day regardless of whether the animal is nocturnal, diurnal or relatively aperiodic with respect to the sleep/wake cycle [39]. Retinal RS cells generally exhibit a reverse pattern [40]. Zebrafish larvae, for example, disassemble all their ribbons during the night [41]. Zebrafish larva also display circadian rhythms in hypocretin neuronal synapses, which vary in number at different times of day [42]. An important observation from this study, consistent with what has been shown in Drosophila, is that different circuits exhibit different rhythms in synapse number. In some circuits, synapses appear to be more numerous in the subjective night, others in the subjective day.

Circadian rhythms in synaptic morphology are reported in the mammalian cortex and hippocampus. In mouse somatosensory (barrel) cortex, excitatory synapses are maximal during the light phase while inhibitory synapses are greatest in the dark phase. Under constant conditions, only changes in inhibitory synapses are observed, consistent with an endogenous rhythm [43]. In the hippocampus and motor cortex dendritic spines are more numerous or larger during the normal active phase (subjective night) [44,45]. While such changes have been ascribed to changes in brain state [8], they instead appear to be regulated by the circadian rhythm in glucocorticoid secretion. For example, adrenalectomy in rats completely abolishes the normal cycling of hippocampal spine density [8]. Similar results are reported in cortex, when the endogenous pattern of corticosterone secretion is disrupted by timed administration of exogenous corticosterone or a suppressor of hypothalamic pituitary adrenal (HPA) axis activity (dexamethasone) [44].

## 4. Mechanisms: Central and Peripheral Clocks

**Figure 1:**
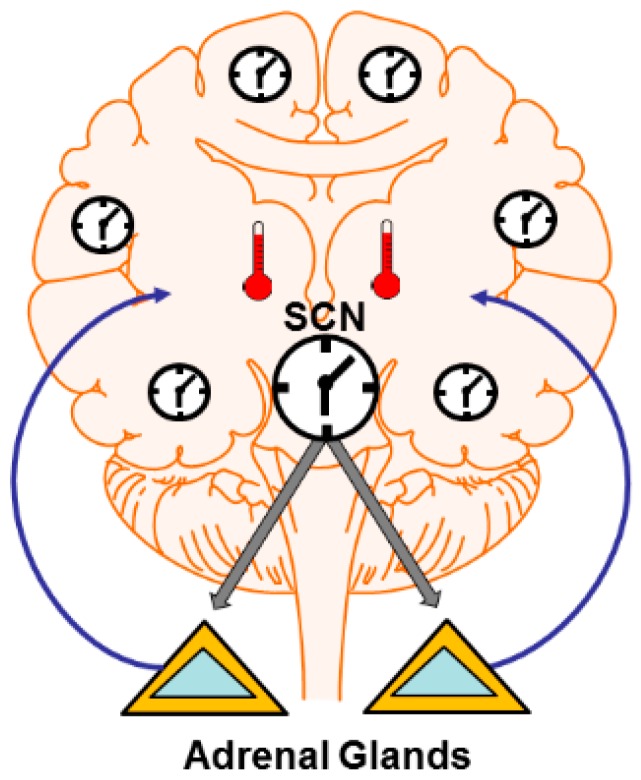
Central and peripheral clocks influence synaptic plasticity. Central clocks like the mammalian suprachiasmatic nucleus (SCN) can impose rhythms in non-clock circuits via several mechanisms. These include rhythms in hormonse and neuromodulator output (e.g., cycles of glucocorticoid release from the adrenal glands) which can alter synapses widely throughout the brain. The SCN also directly drives rhythms in core and brain temperature. Temperature profoundly influences neural function and synaptic plasticity. Temperature may also operate to entrain peripheral clocks in non-SCN neurons. Peripheral clocks themselves can direct plastic changes due to the expression of cannonical clock genes outside central clocks.

Circadian regulation of synaptic plasticity can involve central or peripheral clocks (Figure 1). Central clocks refer to dedicated cells or nuclei that impose rhythmicity on target structures. The mammalian SCN is one example. Peripheral clocks refer to oscillators that express canonical clock genes, are often synchronized by central clocks, but can operate independently from central clocks [46,47]. An example is the peripheral clock in the Drosophila MN5 motor neuron. Central clocks can influence plasticity in three ways. These are the production of 24-h rhythms in brain temperature, hormone and neuromodulator release and GABAergic inhibition. Peripheral clocks may influence plasticity via signaling pathways downstream of cycling clock genes. I discuss these topics below.

### 4.1. Brain Temperature

The biological clock produces 24-h rhythms in core and brain temperature [48]. In endotherms, this involves direct mechanisms of thermogenesis, and in ectotherms, temperature is behaviorally regulated [49]. In both endotherms and ectotherms, temperature can have significant effects on synaptic plasticity [5]. Studies in vitro show that dendritic spines rapidly change their size and shape as a function of temperature. Although these latter studies used large temperature gradients and should be cautiously interpreted, studies in vivo show that normal fluctuations in brain temperature can also alter measures of plasticity. For example, studies in freely moving rodents show that hippocampal EPSPs increase when animals explore novel environments. The latter changes are due to accompanying changes in brain temperature and not “learning” or experience per se [50]. Similar temperature gradients across the day and night have been reported in mammals [51,52].

The effects of temperature may be even more extreme in ectotherms. Temperature gradients as small as ≈8 °C are sufficient to alter synaptic structures in Drosophila [53,54]. These include increased axonal arborization in mushroom body neurons [54] and motor nerve terminals in vivo [53] and neurite extension in vitro [54]. Whether similar temperature gradients exist across the 24-h day is unknown as this has yet to be measured. However, similar gradients in ambient temperature are encountered under natural conditions [55], and may even occur in insects housed under constant ambient temperatures. This is because core temperature tracks motor/muscle activity in small terrestrial ectotherms [54].

How then does temperature influence synaptic plasticity? Many biological processes are profoundly affected by changes in temperature including those that might influence synapse number or strength. In the brain, the Q10 (the change in a biochemical process with a 10° change in temperature) can be higher than in other tissues (a Q10 > 2). This suggests that normal changes in brain temperature can significantly impact neuronal circuits. Indeed, in mammals the normal fluctuation in brain temperature (1–3 °C) is sufficient to alter diverse neural processes encompassing action potential generation, neurotransmitter release, vesicle transport and trans-membrane ionic transport [56]. Therefore, one possibility is direct action on neural enzymes critical for synaptic plasticity. As discussed above a change of ≈8 °C in Drosophila is sufficient to alter axonal morphology. Interestingly, these latter temperature effects are mediated by changes in cAMP, an enzyme critical for many forms of synaptic plasticity [54].

A second potential mechanism is temperature sensitive ion channels that belong to the TRP (transient receptor potential) superfamily. Many TRPs are exquisitely sensitive to temperature (they can have a Q10 > 10), are widely found in vertebrate and invertebrate neurons, and when activated result in an influx of cations into cells [57,58]. The temperature sensitivity of TRPs in structures like the mammalian cortex and hippocampus is not as well understood as in other nervous tissue (e.g., temperature sensitive neurons in the periphery). There is, however, some evidence that they respond to temperature in ways that may influence synaptic plasticity. For example, TRPV4 channels in the hippocampus open in a temperature sensitive manner and their deletion reduces membrane polarization and the ability to induce LTP [59]. Other TRPV channels may instead modulate long-term synaptic depression (LTD); however, temperature-dependence in the latter effects are unclear (reviewed in [60]).

### 4.2. Hormone and Neuromodulator Release

In many animal species a central clock also produces 24-h rhythms in the secretion of hormones and release of neuromodulators, many of which influence neuronal excitability and plasticity [61]. In mammals, these include changes in melatonin and stress hormones (glucocorticoids) (for additional discussion, see [46]). In mammals (including humans) melatonin secretion is maximal during the night and minimal during the light phase. The effects of melatonin appear to be inhibitory on some forms of synaptic strengthening. Nanomolar concentrations of melatonin inhibit rodent hippocampal LTP in vitro [62] and melatonin receptor (MT1 and MT2) double-knock out mice show enhanced LTP and improvements in motor and cognitive tasks [63]. The MT1 and MT2 g-protein coupled receptors are known to inhibit cAMP-activated protein kinase in neurons; an enzyme critical for many forms of LTP. Melatonin may also bind directly to other calcium dependent enzymes involved in LTP, including calmodulin (CaM) and CaM/CaMKII complexes [64]. Moreover, melatonin can reduce neuronal excitability via multiple pathways, including inhibition of nitric oxide synthesis and modulation of GABA and glutamate receptor signaling [64]. This suggests that circadian peaks and troughs in melatonin secretion normally modulate the likelihood of induction or magnitude of some types of plasticity.

Circadian rhythms in circulating glucocorticoid concentrations are a powerful mechanism for altering synaptic strength. Corticosterone can modulate cortical excitability, the amplitude of evoked potentials and α-amino-3-hydroxy-5-methyl-4-isoxazolepropionic acid receptor (AMPAR) trafficking [5]. Small, transient increases in corticosterone can lead to rapid spinogenesis in vivo, which slowly declines over 5 h [65]. These latter findings are consistent with previously reported biphasic effects of glucocorticoids, which are comprised of rapid increases in synaptic efficacy (and/or spine formation) followed by a slower, time-dependent normalization of synapses to baseline levels (for discussion, see [66,67]). These changes in synapses are mediated by action at mineralcorticoid and glucocorticoid receptors, resulting in both fast action at the synapse (e.g., changes in miniature excitatory postsynaptic currents) and longer lasting changes in neurotransmission that reflect genomic changes [68].

### 4.3. Rhythms in GABAergic Inhibition

An intriguing set of findings in Siberian hamsters (*Phodopus sungorus*) indicates that the SCN provides periodic waves of inhibition onto hippocampal circuits. In these rodents, light pulses delivered in a specific manner render the animals completely arrhythmic. The arrhythmia in turn results in severe impairments in hippocampal-dependent spatial and recognition memory [69]. Surprisingly, lesioning the SCN restored normal memory performance, indicating that a disrupted signal emanating from the SCN was involved. The signal appeared to be GABAergic as the deficits could also be reversed by systemic administration of the GABA_A_ antagonist pentylenetetrazol [70]. This concept is further supported by the fact that GABA is the principal neurotransmitter of the SCN, SCN GABA levels oscillate with a 24-h rhythm, and a major target of the SCN is the septum. The septum in turn provides GABAergic input to the hippocampus [70,71]. Intriguingly, the septum also innervates many areas of the cortex (e.g., prefrontal, infralimbic, entorhinal and subiculum) [72,73], therefore, it is possible that similar SCN mediated influences exist outside of the hippocampus.

### 4.4. Peripheral Clocks

Core clock genes are found in a variety of tissues and brain regions outside the classic central clock [46,47]. These form peripheral clocks, which may independently influence plasticity in different circuits. This suggests that clock genes may have roles outside their classic time-keeping functions and/or orchestrate intracellular events that influence the strength or number of synapses. Good examples of these multiple influences can be found in the striatum and the hippocampus. In the striatum, plasticity can occur in dopaminergic synapses. Several dopaminergic genes are direct transcriptional targets of the core clock gene clock; resulting in rhythmic expression of dopamine synthesis and metabolism. The clock proteins Per1 and Per2 are also rhythmically expressed in the striatum and the deletion of Per2 abolishes circadian rhythms in monoamine oxidase A, an enzyme that plays an essential role in dopamine catabolism. Similar roles may exist for other clock genes, including Bmal1 and REV-ERBα (reviewed in [21]). In the hippocampus, the core clock gene Per1 produces rhythmic phosphorylation of CREB; a key enzyme in many forms of transcription-dependent plasticity and hippocampal-based memory. Per1 is also required for rhythmic changes in epigenetic markers that can influence the expression of many plasticity-related transcripts [21].

## 5. Discussion

Circadian rhythms in brain temperature, hormone/neuromodulator concentrations and GABAergic signaling may adjust the gain of different forms of plasticity as a function of circadian time. These central influences likely work in concert with peripheral clocks that modulate the response to central influences [46] and also independently control cellular processes that impact plasticity. The functional consequences of such rhythmicity need to be explored, but this could be adaptive in several ways. First, it ensures that an organism’s nervous system is optimized to encode experience during wakefulness. Second, it may separate the induction and consolidation of plastic changes—which are both energetic processes—across the 24-h day. The latter process would then be expected to coincide with sleep. Indeed, sleep (or the “inactive” phase) has been linked with various forms of memory consolidation and persistent forms of plasticity [74,75]. Third, it may provide a means of globally adjusting synaptic strength (a process known as synaptic scaling or homeostatic plasticity [76]) that offsets Hebbian plasticity triggered by experience. A form of scaling has been hypothesized to occur principally in sleep [77,78], but outputs of central and peripheral clocks could instead govern this process.

A reasonable question at this point is: what then is the role of sleep in synaptic plasticity? This question has been vigorously debated for decades, and yet no simple answer exists. Over the decades, sleep has been variously proposed to strengthen, stabilize or weaken synapses (reviewed in [74,79,80]). These ideas themselves have an impressive rhythmicity, reappearing in various guises over and over again, buoyed by periodic waves of supportive findings (for discussion, see [4,5]. More recently, the idea that sleep globally weakens synapses has been proposed as an explanation for why we sleep [77,78]. Some findings are consistent with the view [78], but others are not (for discussion, see [5,6]). For example, the effects of sleep on plasticity are highly dependent on the type of circuit under examination and when in the 24-h day measurements of plastic change are made [4,5]. These latter observations are particularly telling because they strongly suggest the influence of biological clocks.

**Figure 2:**
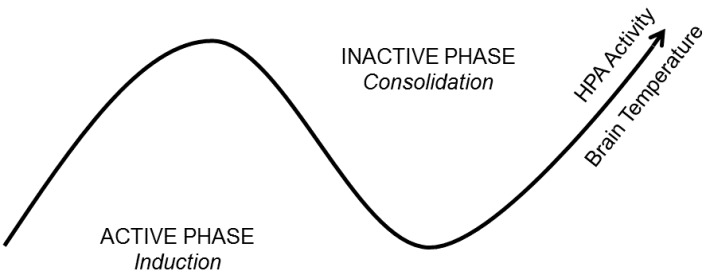
A State-Clock Model (SCM) of sleep and circadian regulation of synaptic plasticity. According to the SCM, biological clocks produce circuit-specific, 24-h rhythms in synaptic efficacy and morphology. It proposes that global synaptic changes observed across sleep and wake are driven by clocks and not brain state. This ensures that an organism’s nervous system is optimized to encode experience during wakefulness and separates the induction and consolidation of plastic changes across the 24-h day. The latter process would then be expected to coincide with brain states conducive for consolidation (sleep). HPA = Hypothalamic-Pituitary-Adrenal axis.

Based on these and similar observations, a “State-Clock” model (SCM) was proposed (Figure 2), according to which outputs of the biological clock produce circuit-specific, 24-h rhythms in synaptic efficacy and morphology [4]. In contrast to other theories [78], it proposes that global synaptic changes observed across sleep and wake are driven by clocks and not brain state. The SCM thus may account for some of the variability in synaptic changes reported after sleep. For example, it explains why evidence of global synaptic weakening after sleep is not reported in carnivores with weak or absent circadian organization [4]. It also accounts for the observation that evidence of global synaptic weakening in rodents in vivo is typically reported when measurements are made after long periods of sleep (e.g., 6 or 12 h) [5]. However, when conducted this way, measurements made before and after sleep occur at very different phases of the circadian cycle.

In the SCM sleep principally acts to consolidate the effects of waking experience by transforming labile plastic changes into more persistent forms. This likely involves a shift from mRNA transcription to translation; a process shown to be sleep-dependent in species with weak or strong circadian rhythms [81,82]. As consolidation reinforces or stabilizes a plastic change induced in prior wake, it does not require that all synapses necessarily be weaker (or stronger) after sleep. This is because the sign of plastic change is determined by the kinds of waking experience that precede sleep (i.e., waking experience can weaken or strengthen synapses) [5]. This function may work in conjunction with circadian rhythms, but does not require them. For example, the circadian rhythm in glucocorticoid secretion not only induces dendritic spine formation during the active phase, but also stabilizes newly formed spines during the inactive phase. These changes in spines are likely to play an important role in the consolidation of experience [44].

## 6. Conclusions

The role of central and peripheral clocks in synaptic plasticity is relatively unexplored. Not surprisingly, there are a number of important future directions and unanswered questions. One important future direction is to test predictions of the SCM. One way would be to remove circadian rhythms in hormone secretion or temperature. For example, if circadian rhythms in glucocorticoid secretion play an essential role, then eliminating such rhythms (via adrenalectomy [83]) should eliminate reported global sleep-wake differences in synaptic markers and plasticity [78]. There may also be ways to “clamp” core temperature in mammals [48], which would also eliminate the influence of circadian temperature cycles on synaptic efficacy and morphology. In mammals, this can be done via implantation of thermocouples to control the activity of temperature-sensitive neurons in the hypothalamus that regulate global brain temperature [48]. Alternatively, discrete regions of cortex or hippocampus could be cooled or warmed using a similar approach. A second important test would be to dissociate circadian rhythms from the sleep-wake cycle using forced-desynchrony protocols [84]. A prediction of the SCM is that global synaptic changes will remain in phase with the circadian cycle and not depend on changes in brain state. This prediction has been partially borne out in human studies of the electroencephalogram (EEG) [85]. In this study, the slope of EEG slow-waves (which is considered a measure of synaptic strength [86]) was shown to vary with circadian phase. In some cases, the circadian influence was equal or greater to the influence of sleep pressure. Inducible deletion of clock genes both centrally and peripherally would also provide an interesting test of the role of rhythms in plasticity. This would avoid potential confounds that arise from embryonic deletions, as it has been shown that clock genes may govern important aspects of brain development independent of their circadian time-keeping roles [87,88].

There are also a number of interesting questions that must await future investigation. One puzzling aspect of the SCN is that its outputs can preserve their sign regardless of whether species are diurnal or nocturnal. The secretion of melatonin for example is always maximal during the dark period even in nocturnal rodents. This seems counter-intuitive as melatonin appears to inhibit neural excitation and synaptic potentiation; processes that are generally promoted during the rodent active phase. The cycle of melatonin secretion also seems to work at cross-purposes with rhythms of brain temperature and glucocorticoid secretion that promote global increases in synaptic strength. It is possible that this may one day be explained by regional brain differences in the response to these factors, but to date this seeming paradox is unexplained. Another interesting question concerns peripheral clocks. Only a handful of studies have explored the role of core clock genes in synaptic plasticity. Consequently, we know very little about how they might influence receptor trafficking, the transcription of plasticity related mRNAs, protein synthesis and other essential processes in synaptic remodeling. A related question is how do central and peripheral clocks interact? As proposed by Mohawk et al. [9], core temperature rhythms driven by the SCN reset and entrain peripheral clocks in organs. Could a similar relationship exist between central clocks and peripheral clocks in extra-SCN neurons? It will also be important to re-examine the relationship between mammalian network events that occur during sleep (e.g., hippocampal sharp-waves and ripples), plasticity and circadian phase. Although these events can occur at the millisecond scale, they may be modulated by the slower envelope of changes in brain temperature and hormone release. A final important question to address is the functional significance of circadian rhythms in plasticity. Although one can, as I have, present plausible explanations for why this should occur, the fact remains that we know very little about how such rhythms impact essential neural function.

## Abbreviations

The following abbreviations are used in this manuscript:

AMPAR	α-amino-3-hydroxy-5-methyl-4-isoxazolepropionic acid receptor
Cam	calmodulin
LTP	Long-term potentiation
LTD	Long-term depression
SCN	Suprachiasmatic Nucleus
SCM	State-Clock Model
LNv	Lateral-Ventral Neuron
LD	Light-Dark
DD	Dark-Dark
TRP	transient receptor potential
